# Associations between psychedelic use and migraine history in Swedish twins

**DOI:** 10.1177/02698811261449385

**Published:** 2026-05-11

**Authors:** Otto Simonsson, Sunjuri Sun, Laura W. Wesseldijk, Fredrik Ullén, Walter Osika, Miriam A. Mosing

**Affiliations:** 1 Department of Neurobiology, Care Sciences and Society, Karolinska Institutet, Stockholm, Sweden; 2 Department of Global Public Health, Karolinska Institutet, Stockholm, Sweden; 3 Department of Neuroscience, Karolinska Institutet, Stockholm, Sweden; 4 Department of Psychiatry, Amsterdam UMC, University of Amsterdam, the Netherlands; 5 Department of Cognitive Neuropsychology, Max Planck Institute for Empirical Aesthetics, Frankfurt am Main, Germany; 6 Department of Medical Epidemiology and Biostatistics, Karolinska Institutet, Stockholm, Sweden; 7 Melbourne School of Psychological Sciences, Faculty of Medicine, Dentistry, and Health Sciences, University of Melbourne, VIC, Australia

**Keywords:** psychedelics, psilocybin, LSD, migraine, twins

## Abstract

**Background:**

While psychedelics have shown initial promise in the treatment of migraine, experimental studies have relied on small and homogenous samples, which limit the reliability and generalizability of findings. These limitations underscore the complementary value of other research designs that leverage larger and more representative samples.

**Methods:**

This cross-sectional study included three cohorts of twins from the Swedish Twin Registry and evaluated associations between psychedelic use and migraine history.

**Results:**

In this study, 50,726 twins answered questions related to the use of psychedelics. There were 1287 twins who reported psychedelic use, of whom 420 were monozygotic twins. While 271 twin pairs were discordant on psychedelic use, 40 twin pairs (16 male, 24 female) were discordant on both psychedelic use and migraine history. When restricting the analyses to monozygotic twins in the between-within logistic regression model, the between-pair association was significant, with pairs in which at least one twin reported psychedelic use showing lower odds of migraine history (adjusted odds ratio (aOR) = 0.50, *p* = 0.041). The within-pair association was also significant, with twins who reported psychedelic use showing lower odds of migraine history compared to their co-twin (aOR = 0.38, *p* = 0.008). Notably, subgroup analyses showed that these results were broadly the same when only males were included in the models, but no significant associations were observed in female-only models.

**Conclusions:**

The findings from this twin study suggest that psychedelics may be linked to a lower likelihood of migraine, with potential differences by sex. This warrants further investigation and highlights the importance of sex-specific analyses in future studies.

## Introduction

Migraine is a neurological disorder characterized by headache episodes of varying degrees of severity. It can cause debilitating pain and is a leading cause of disability, particularly among females (Steiner et al., 2020). The estimated prevalence of migraine worldwide is approximately 15%, with two to three times higher prevalence in females compared to males, which may reflect differential effects of sex hormones (Steiner and Stovner, 2023; Vetvik and MacGregor, 2017). While there are both preventive and acute pharmacological treatments available, many of these have limited efficacy and considerable side effects (Eigenbrodt et al., 2021; Johnston et al., 2022; Lampl et al., 2023). It is therefore important to investigate alternative pharmacotherapies for migraine.

Psychedelic substances such as psilocybin and lysergic acid diethylamide (LSD) have been shown to be promising in the treatment of migraine (Schindler, 2023; Schindler et al., 2021). For example, in both randomized and non-randomized, double-blind, placebo-controlled trials among adults with a migraine diagnosis, psilocybin administration has been associated with a reduction in migraine frequency, though evidence for drug-specific effects remains mixed (Schindler et al., 2021, 2025a). Other psychedelic trials involving adults with related neurological disorders (e.g. cluster headache) have found similar results, but the relatively small and homogenous samples used in these studies limit the reliability and generalizability of the findings, as well as the ability to investigate sex-specific differences (Bhanot et al., 2023; Rucker et al., 2024; Schindler et al., 2022, 2024). These limitations underscore the complementary value of other research designs that leverage larger and more representative samples.

Recent epidemiological studies have partially replicated findings from experimental studies on the effects of psychedelics on migraine and other headache disorders (Andersson et al., 2017; Bjurenfalk et al., 2025; Cavarra et al., 2024; Rusanen et al., 2022; Smedfors et al., 2024). For instance, in a cross-sectional study using a British cohort sample, psychedelic use was associated with lower odds of having frequent bad headaches. Further subgroup analyses in the same study showed that psychedelic use was only associated with lower odds of having frequent bad headaches among females, but it is possible that the null findings in the subgroup analyses on males were due to a lack of statistical power (Bjurenfalk et al., 2025). It is also worth noting that the study design was susceptible to familial confounding and therefore should be replicated using more rigorous designs (e.g. co-twin control models) that can provide support for causal inferences.

In this study, we utilized three cohorts of twins from the Swedish Twin Registry (STR) – the Child and Adolescent Twin Study in Sweden (CATSS), the Young Adult Twins in Sweden Study (YATSS), and the Study of Twin Adults: Genes and Environment (STAGE) – to evaluate associations between psychedelic use and migraine history. We also used twin modeling to test whether any observed associations were consistent with a causal relationship and replicated all analyses in subsamples stratified by sex to investigate potential sex-specific associations.

## Materials and methods

### Study population

This study combined three Swedish twin cohorts from the STR that all included data on self-reported psychedelic use and migraine history: CATSS (born 1992–2005), YATSS (born 1985–1992), and STAGE (born 1958–1985; Lichtenstein et al., 2006; Zagai et al., 2019). In CATSS, twins were asked about psychedelic use and migraine history at age 15, while these questions were asked in adult age for the YATSS and STAGE cohorts. Across all 3 cohorts, 50,726 participants answered questions about past use of psilocybin or LSD. Among these, 47,452 also answered questions about migraine history. Ethical approval for this study was granted by the Swedish Ethical Review Authority (reference number: 2024-00521-01). Informed consent was obtained from all participants. The study followed the Strengthening the Reporting of Observational Studies in Epidemiology reporting guidelines. Data were analyzed from January 2025 to August 2025.

## Measures

### Drug use

Participants were asked about past use of various drugs. Participants who indicated that they had never used psilocybin or LSD were coded as 0 for psychedelic use, while the participants who reported previous use of psilocybin or LSD were coded as 1. Other substance use items included alcohol, tobacco, cannabis, stimulants, sedatives, opioids, and performance-enhancers. No question was available in YATSS about ever using tobacco (i.e. cigarettes or snuff), but there were lifetime use threshold questions (i.e. more than 100 cigarettes in lifetime, more than 5 boxes of snuff in lifetime) that were used instead (Supplemental eTable 1). There was a question about inhalants in CATSS, but this item was not available in YATSS or STAGE and was therefore not included in this study. The responses that were unspecific (e.g. “don’t know,” “don’t want to answer”) were coded as missing values.

### Migraine history

Participants in CATSS were asked whether they had ever had a migraine diagnosis (“Have you ever had any of the following diagnoses? Migraine”). Participants in YATSS and STAGE were asked whether they have or have ever had migraine (“Do you have or have you ever had. . . Migraine”). Participants who indicated no migraine at present or earlier in life were coded as 0 for migraine history, while the participants who reported a current or previous history of migraine were coded as 1. The responses that were unspecific (e.g. “don’t know,” “don’t want to answer”) were coded as missing values.

### Statistical analyses

Associations between psychedelic use and migraine history were evaluated by statistical models run in Stata version 18 (StataCorp LLC, College Station, TX, USA). Model 1 used a logistic regression with robust standard errors for clustering to estimate associations between psychedelic use and migraine history. While a logistic regression can provide general population-level association estimates, it does not control for genetic and family environmental confounding, which limits the ability to make causal inferences from the results. Because monozygotic twins have essentially identical DNA and also share their rearing environment, using models that instead compare differences within monozygotic twin pairs can control for genetic and family environmental confounding, effectively revealing associations that are not due to familial confounding. Such an approach can evaluate whether an observed association likely reflects a causal effect rather than shared familial influences (Gonggrijp et al., 2023; McGue et al., 2010; Sjölander et al., 2012). Model 2 therefore used a between-within (random-effects) logistic regression in monozygotic twins, decomposing psychedelic use into between-pair (mean use) and within-pair (deviation from mean) components. A random intercept accounted for twin-pair clustering, enabling estimation of associations at both the family and individual levels while controlling for shared genetic and environmental factors.

All models adjusted for past use of alcohol, tobacco, cannabis, stimulants, sedatives, opioids, and performance-enhancing drugs; Models 1 and 2 also controlled for cohort (i.e. CATSS, YATSS, STAGE) and sex. Further analyses in subsamples stratified by sex were conducted to investigate potential sex-specific associations, and sex × psychedelic use interactions on the within-pair estimates were tested to evaluate whether these differences were statistically significant. In each analysis, observations with missing values in any of the variables included in the model were excluded from that specific analysis. We assessed multicollinearity using variance inflation factors (VIF), ensuring that no covariates exceeded a VIF threshold of 10. In all tests a two-sided *p* < 0.05 was used as significance threshold.

As sensitivity analyses, we used a fixed-effects logistic regression restricted to monozygotic twin pairs to estimate within-pair associations, effectively controlling for all shared familial confounders (i.e. shared genetic and rearing environmental effects). Because only discordant monozygotic twin pairs were included in the fixed-effects logistic regression, it had less statistical power than the between-within (random-effects) logistic regression. However, the fixed-effects logistic regression provides a more conservative and robust estimate of associations, which makes it useful as part of sensitivity analyses. As additional sensitivity analyses, covariates were collapsed across all models into substance-aggregated categories used in prior twin studies of psychedelic use (Simonsson et al., 2024): (1) alcohol and tobacco; and (2) cannabis, stimulants, sedatives, opioids, and performance-enhancing substances.

## Results

### Descriptive statistics

Table 1 shows descriptive statistics of twins included in the analyses. Of the 50,726 twins who provided information on psychedelic use, 47,010 (93%) had complete data on migraine history and all covariates. There were 1287 twins who reported psychedelic use (i.e. psilocybin or LSD; 2.5% of the sample), of whom 420 were monozygotic twins. Notably, 271 twin pairs were discordant on psychedelic use, while 40 twin pairs (16 male, 24 female) were discordant on both psychedelic use and migraine history (see Supplemental eTable 2 for descriptive statistics by cohort).

**Table 1.** Descriptive statistics.

Characteristic	Past use of psychedelics	
	Yes (*N* = 1287)	No (*N* = 49,439)
Migraine history		
Yes	219	8982
No	924	37,327
Past use of alcohol		
Yes	1276	37,728
No	11	11,648
Past use of tobacco		
Yes	486	13,705
No	792	35,366
Past use of cannabis		
Yes	1231	5614
No	54	43,806
Past use of stimulants		
Yes	1055	1289
No	232	48,150
Past use of sedatives		
Yes	873	1734
No	407	47,673
Past use of opioids		
Yes	892	3251
No	395	46,118
Past use of performance-enhancers		
Yes	714	165
No	572	48,271
Sex		
Male	736	21,469
Female	551	27,970
Zygosity		
MZ twins	420	17,617
MZ twin pairs discordant on psychedelic use	271	271
MZ twin pairs discordant on both psychedelic use and migraine history	40	40
Male MZ twin pairs discordant on both psychedelic use and migraine history	16	16
Female MZ twin pairs discordant on both psychedelic use and migraine history	24	24

*Note.* Due to missing data, total numbers for each category may not add up to total number of twins who reported past use of psychedelics within each cohort. MZ: Monozygotic.

### Inferential statistics

#### Associations between psychedelic use and migraine history

In the logistic regression model, psychedelic use was associated with lower odds of migraine history (Adjusted odds ratio (aOR) = 0.76, *p* = 0.017; see Model 1 in Table 2). Further subgroup analyses showed that these results were broadly the same when only males were included in the model (aOR = 0.70, *p* = 0.032), but no significant association was observed in the model that included only females (*p* > 0.05; see Supplemental eTable 3).

**Table 2.** Model estimates – Psychedelic use and migraine history.

Exposure	Model 1			Model 2				
	Logistic regression			Between-pair		Within-pair		
	aOR (95% CI)	*p*	*n*	aOR (95% CI)	*p*	aOR (95% CI)	*p*	*n* (pairs, discordant)
Psychedelic use	0.76 (0.60, 0.95)	0.017	47,010	0.50 (0.26, 0.97)	0.041	0.38 (0.19, 0.78	0.008	16,771 (10,065, 239)

*Note.* Adjusted odds ratios (aORs) and 95% confidence intervals (CIs), as well as *p*-value (*p*) and number of observations (*n*), are reported for each model. *n* (pairs, discordant) under Model 2: individuals in the analytic model (twin pairs, exposure‑discordant pairs after exclusion for missing outcome and covariates). Model 1 is a logistic regression; Model 2 is a between-within (random-effects) logistic regression. All models estimated the association between the exposure (lifetime psychedelic use) and the outcome (migraine history) while controlling for past use of alcohol, tobacco, cannabis, stimulants, sedatives, opioids, and performance-enhancers, as well as cohort (i.e. CATSS, YATSS, STAGE) and sex. CATSS: Child and Adolescent Twin Study in Sweden; YATSS: Young Adult Twins in Sweden Study; STAGE: Study of Twin Adults: Genes and Environment.

#### Between-within associations between psychedelic use and migraine history in monozygotic twins

Restricting the analyses to monozygotic twins in the between-within logistic regression model, the between-pair association was significant, with pairs in which at least one twin reported psychedelic use showing lower odds of migraine history (aOR = 0.50, *p* = 0.041). The within-pair association was also significant, with twins who reported psychedelic use showing lower odds of migraine history compared to their co-twin (aOR = 0.38, *p* = 0.008; see Model 2 in Table 2). Further subgroup analyses showed that these results were broadly the same when only males were included in the model (between-pair aOR = 0.39, *p* = 0.047; within-pair aOR = 0.15, *p* < 0.001), but no significant associations were observed in the model that included only females (*ps* > 0.05; see Supplemental eTable 2). Sensitivity analyses showed estimates in the same direction as the primary analyses, though with wider confidence intervals (see Supplemental eTable 4). Notably, interactions between sex and psychedelic use in co-twin control analyses appeared to indicate a stronger protective association in men than in women (*ps* = 0.039–0.069).

## Discussion

This cross-sectional study investigated the associations between psychedelic use and migraine history in three large Swedish twin cohorts. In covariate-adjusted models, psychedelic use was associated with lower odds of migraine history, even in analyses that accounted for familial confounding. These results build on preliminary evidence from experimental studies and provide additional support for a potential protective relationship between psychedelics and migraine.

If psychedelic substances such as psilocybin and LSD have a causal protective effect on migraine, it is plausible that the mechanism may be at least partially neurobiological. For instance, both migraine pathophysiology and the effects of psychedelics converge on the serotonin system, while psychedelics are chemically similar to certain migraine treatments, such as dihydroergotamine and triptans (Schindler, 2023), and may have anti-inflammatory effects (Flanagan and Nichols, 2018). Previous research on individuals who reported using psychedelics for self-treatment suggests that various doses and dosage regimens were considered effective for both prophylactic and acute treatment of migraine (Andersson et al., 2017). Other research also suggests that the effects of psychedelics on headache frequency in adults with migraine or other headache disorders may not be related to the subjective experience during the acute effects (Schindler et al., 2025b), which contrasts with findings on the effects of psychedelics on mental health (Yaden and Griffiths, 2020). Future studies should further investigate the mechanism underlying the potential causal protective effect of psychedelics on migraine.

Notably, subgroup analyses showed sex-specific associations between psychedelic use and migraine history, which could have different complementary explanations. For example, there are migraine subtypes that are relatively unique to females (e.g. menstrual migraine) and these may not respond to psychedelics in the same way as other migraine types that occur in both sexes. Because participants were not asked to distinguish between migraine subtypes, it is possible that there may have been conflation of specific migraine subtypes, which could explain the sex-specific associations in this study. Another possibility is that the observed sex-specific associations reflect general or migraine-specific sex differences in response to psychedelics. These could, in turn, be influenced by hormonal factors among females relating to, for example, menstrual cycle stages (e.g. menstruation) or developmental stages (e.g. menopause; Shadani et al., 2024). Future studies should investigate the effects of psychedelics on specific migraine subtypes, as well as sex-specific differences in response to psychedelics and the potential role of sex hormones.

## Strengths and limitations

This study has several strengths, including the use of a large, population-based twin sample, the co-twin control design which accounts for shared genetic and environmental confounding, and the relatively high levels of data completeness among twins who provided information on psychedelic use, reducing the likelihood of bias due to missing data. There are, however, limitations that should be considered when interpreting the results. First, due to the exploratory nature of the study, no adjustments were applied for multiple statistical tests, potentially increasing the risk of Type I error. Second, the cross-sectional study design makes it impossible to exclude reverse causality. It is, for example, conceivable that the twin without a migraine history in the first place was more likely to use psychedelics than their co-twin with a migraine history. Previous research suggests that individuals with migraine are less likely to use illicit or licit substances, which provides support for the possibility of reverse causality (van den Hoek et al., 2024). Third, participants were asked general questions about psychedelic use and migraine history. No data were collected on other types of psychedelics (e.g. mescaline, ayahuasca), on exposure metrics related to psychedelic use (e.g. frequency, dose), on motivation for use (e.g. self-treatment, recreational), or on migraine subtypes (e.g. menstrual migraine), which could have provided nuance to the observed associations between psychedelic use and migraine history. Fourth, the use of self-report measures may have led to biased responses. The outcome variable, for instance, was based on self-reported history of migraine rather than clinical verification through medical records, which may have introduced recall bias or misclassification, especially among individuals with infrequent episodes. It is therefore possible that the sex-specific findings could partly be explained by the misclassification of even a small number of individuals with conditions more prevalent in men, such as cluster headaches (Fischera et al., 2008). Fifth, among the participants who reported psychedelic use, a large proportion also reported use of other drugs (e.g. cannabis, stimulants, sedatives), which makes it challenging to isolate the specific associations with psychedelic use. Future studies should use longitudinal twin designs, collect data on other types of psychedelics and exposure metrics related to psychedelic use, and investigate associations with specific migraine subtypes, possibly using medical diagnosis as outcomes through linkage with healthcare registry data.

## Conclusions

While psychedelics have shown initial promise in the treatment of migraine, experimental studies to date have relied on small and homogenous samples, which limit the reliability and generalizability of the findings. These limitations underscore the complementary value of other research designs that leverage larger and more representative samples, especially those that can control for genetic and family environmental confounding. In this cross-sectional study of twins from three Swedish cohorts, we investigated associations between psychedelic use and migraine history. In covariate-adjusted models, psychedelic use was associated with lower odds of migraine history, even in analyses that accounted for familial confounding. Notably, subgroup analyses showed that these results were broadly the same when only males were included in the models, but no significant associations were observed in female-only models. These findings build on preliminary evidence from experimental studies, strengthen the hypothesis of a causal relationship, and highlight the potential importance of sex-specific analyses in future studies. However, in light of the study limitations, the findings should be interpreted with caution until replicated, ideally with longitudinal data, more detailed information on psychedelic use and migraine subtypes, and objective measures such as medical diagnoses in the healthcare system.

## Supplemental Material

sj-docx-1-jop-10.1177_02698811261449385 – Supplemental material for Associations between psychedelic use and migraine history in Swedish twins Supplemental material, sj-docx-1-jop-10.1177_02698811261449385 for Associations between psychedelic use and migraine history in Swedish twins by Otto Simonsson, Sunjuri Sun, Laura W. Wesseldijk, Fredrik Ullén, Walter Osika and Miriam A. Mosing in Journal of Psychopharmacology
