# Supplementary material for: Associations between psychedelic use and migraine history in Swedish twins
Source: J Psychopharmacol. 2026 May 11;40(5):831–6. doi: 10.1177/02698811261449385 (PMC13310263; doi:10.1177/02698811261449385)
Supplement: sj-docx-1-jop-10.1177_02698811261449385 – Supplemental material for Associations between psychedelic use and migraine history in Swedish twins [file sj-docx-1-jop-10.1177_02698811261449385.docx]

**Supplement**

| eTable 1: Survey items by cohort and substance category | | | |
| --- | --- | --- | --- |
| CATSS | YATSS | STAGE | Substance Category |
| *LSD, "acid"* | *LSD* | *LSD* | *Psychedelics* |
| *Magic mushrooms, Psilocybin* | *Hallucinogenic fungi (psilocybin, psilocin)* | *Mushroom* | *Psychedelics* |
| *Beer (excluding lightbeer), or strong cider* | *Have you ever drunk alcohol?* | *Have you ever in your life drunk beer, wine or hard liquor?* | *Alcohol* |
| *Wine, alcopops, shots, drinks or hard liquor/schnapps (e.g. brandy, moonshine, vodka, gin, cognac, whisky, liqueur, punch)* | *…* | *…* | *Alcohol* |
| *Do you smoke cigarettes?*  *No, I have never smoked* | *Have you smoked more than 100 cigarettes in your entire life?* | *Have you ever smoked or used snuff? No, not even tried* | *Tobacco* |
| *Do you use snuff?*  *No, I have never used snuff* | *Have you used more than 5 boxes of snuff during your lifetime?* | *…* | *Tobacco* |
| *Cannabis, marijuana or hashish* | *Marijuana or Hasch* | *Marijuana* | *Cannabis* |
| *…* | *…* | *Hashish* | *Cannabis* |
| *Amphetamine, khat* | *Amphetamine* | *Amphetamine* | *Stimulants* |
| *Cocaine, crack* | *Cocaine* | *Cocaine* | *Stimulants* |
| *Ecstasy* | *Ecstasy* | *Ecstasy* | *Stimulants* |
| *…* | *Methylphenidate (Ritalina, Rilatin)* | *Methylphenidate (Ritalina, Rilatin)* | *Stimulants* |
| *Sleeping pills, tranquilizers (te.g. Rohypnol, Nitrazepam, Sobril, Stesolid, Valium, Xanor, Imovane, Stilnoct)* | *Rohypnol, Roppar* | *Rohypnol, Roppar* | *Sedatives* |
| *…* | *Other sleeping or sedative agents of benzodiazepine-type (eg Sobril, Xanor, Stilnoct, Stesolid, Zopiclone, etc.)* | *Other soporific or other tranquillizer like benzodiazepines* | *Sedatives* |
| *Heroin, smoke heroin or opium* | *Opium, Morphine or Heroin* | *Opium* | *Opioids* |
| *Morphine, dolcontin, ketogan, ketodur, methadone* | *…* | *Morphine* | *Opioids* |
| *…* | *…* | *Heroin* | *Opioids* |
| *Painkillers (te.g. Distalgesic, Citodon, Kodein, Treo Comp)* | *Citodon* | *Citodon* | *Opioids* |
| *…* | *Codeine* | *Codein* | *Opioids* |
| *…* | *Treo-Comp* | *Treo* | *Opioids* |
| *GHB* | *GHB* | *GHB* | *Performance-enhancers* |
| *Anabolic Steroids* | *Androgenic Anabolic Steroids (AAS)* | *Steroids* | *Performance-enhancers* |
| *…* | *Growth hormone* | *Growth hormone* | *Performance-enhancers* |
| Note: Participants reporting never having used any of the drugs within each category were coded as 0, whereas those who reported past use of any of the drugs within each category were coded as 1. | | | |

| eTable 2. Descriptive statistics by cohort | | | | | | |
| --- | --- | --- | --- | --- | --- | --- |
|  | CATSS | | YATSS | | STAGE | |
|  | Past use of psychedelics | | | | | |
|  | Yes (N=680) | No (N=19,469) | Yes (N=246) | No (N=5,884) | Yes (N=361) | No (N=24,086) |
| **Migraine History** |  |  |  |  |  |  |
| Yes | 89 | 2,139 | 48 | 1,247 | 82 | 5,596 |
| No | 529 | 16,017 | 120 | 3,013 | 275 | 18,297 |
| **Past Use of Alcohol** |  |  |  |  |  |  |
| Yes | 670 | 8,425 | 245 | 5,743 | 361 | 23,560 |
| No | 10 | 11,029 | 1 | 138 | 0 | 481 |
| **Past Use of Tobacco** |  |  |  |  |  |  |
| Yes | 277 | 5,527 | 198 | 2,304 | 344 | 5,874 |
| No | 401 | 13,903 | 47 | 3,574 | 11 | 17,889 |
| **Past Use of Cannabis** |  |  |  |  |  |  |
| Yes | 663 | 475 | 231 | 1,654 | 337 | 3,485 |
| No | 15 | 18,975 | 15 | 4,230 | 24 | 20,601 |
| **Past Use of Stimulants** |  |  |  |  |  |  |
| Yes | 662 | 88 | 150 | 340 | 243 | 861 |
| No | 18 | 19,381 | 96 | 5,544 | 118 | 23,225 |
| **Past Use of Sedatives** |  |  |  |  |  |  |
| Yes | 637 | 300 | 95 | 168 | 141 | 1,266 |
| No | 36 | 19,137 | 151 | 5,716 | 220 | 22,820 |
| **Past Use of Opioids** |  |  |  |  |  |  |
| Yes | 657 | 779 | 77 | 141 | 158 | 2,331 |
| No | 23 | 18,690 | 169 | 5,743 | 203 | 21,755 |
| **Past Use of Performance-Enhancers** |  |  |  |  |  |  |
| Yes | 633 | 13 | 31 | 27 | 50 | 125 |
| No | 46 | 19,453 | 215 | 5,857 | 311 | 23,961 |
| **Sex** |  |  |  |  |  |  |
| Male | 389 | 8,706 | 135 | 2,276 | 212 | 10,487 |
| Female | 291 | 10,763 | 111 | 3,608 | 149 | 13,599 |
| Note: Due to missing data, total numbers for each category may not add up to total number of twins who reported past use of psychedelics within each cohort. | | | | | | |

| eTable 3. Model Estimates – Psychedelic Use and Migraine History | | | | | | | | |
| --- | --- | --- | --- | --- | --- | --- | --- | --- |
|  | Migraine History | | | | | | | |
|  | Model 1 | | | Model 2 | | | | |
|  | Logistic Regression | | | Between-Pair | | Within-Pair | |  |
|  | aOR (95% CI) | p | n | aOR (95% CI) | p | aOR (95% CI) | p | n (pairs, discordant) |
| Males | 0.70 (0.50, 0.97) | .032 | 20,517 | 0.39 (0.15, 0.99) | .047 | 0.15 (0.05, 0.44) | <.001 | 6,978 (4,363, 112) |
| Females | 0.81 (0.59, 1.12) | .199 | 26,493 | 0.54 (0.20, 1.44) | .219 | 0.86 (0.31, 2.40) | .780 | 9,793 (5,702, 127) |
| Note: Adjusted odds ratios (aORs) and 95% confidence intervals (CIs), as well as p-value (p) and number of observations (n), are reported for each model. n (pairs, discordant) under Model 2 = individuals in the analytic model (twin pairs, exposure‑discordant pairs after exclusion for missing outcome and covariates). Model 1 is a logistic regression; Model 2 is a between-within (random-effects) logistic regression. All models estimated the association between the exposure (lifetime psychedelic use) and the outcome (migraine history) while controlling for past use of alcohol, tobacco, cannabis, stimulants, sedatives, opioids, and performance-enhancers, as well as cohort (i.e., CATSS, YATSS, STAGE). | | | | | | | | |

| eTable 4: Model Estimates – Psychedelic Use and Migraine History | | |
| --- | --- | --- |
|  | Sensitivity Analyses | |
| Sample | aOR (95% CI) | p |
| Both Sexes | 0.42 (0.16, 1.14) | .087 |
| Males | 0.11 (0.01, 0.89) | .039 |
| Females | 0.72 (0.21, 2.46) | .600 |
| Note: Adjusted odds ratios (aORs) and 95% confidence intervals (CIs) are reported for each model. All models estimated the association between the exposure (lifetime psychedelic use) and the outcome (migraine history) while controlling for past use of alcohol, tobacco, cannabis, stimulants, sedatives, opioids, and performance-enhancers. All models are fixed-effects logistic regressions, estimating within-pair associations. | | |
